# Antidiabetic, Antioxidant and Anti-Inflammatory Activities of Residual Aqueous Fraction of *Ethulia conyzoides* in Induced Type 2 Diabetic Rats

**DOI:** 10.21315/tlsr2023.34.1.8

**Published:** 2023-03-31

**Authors:** Helen Omasan Okotie, Tayo Micheal Anjuwon, Okwubenata Lilian Okonkwo, Danladi Amodu Ameh, Dorcas Bolanle James

**Affiliations:** 1 Department of Biochemistry, Ahmadu Bello University, Zaria, Nigeria; 2 Immunology Unit, Department of Internal Medicine, Faculty of Clinical Sciences, College of Medicine, Ahmadu Bello University Teaching Hospital, Zaria, Nigeria

**Keywords:** Antidiabetics, Antioxidant, Cytokines, *Ethulia conyzoides*, Type 2 Diabetes

## Abstract

Oxidative stress and inflammation have been proven to be implicated in the pathogenesis of type 2 diabetes. Recent studies showed that *Ethulia conyzoides* had *in-vitro* antioxidant activity. This study investigated the *in-vivo* antidiabetic, antioxidant, and anti-inflammatory potential of the residual aqueous fraction of *Ethulia conyzoides* in type 2 diabetic-induced male *Wistar* rats. Sub-acute antidiabetic studies were done with varying doses (100, 200, and 400 mg/kg body weight) of residual aqueous fraction for 21 days. Blood glucose levels, serum insulin, and in vivo antioxidant and pro-inflammatory cytokines—tumour necrosis factor-α (TNF-α) and interleukin-1β (IL-1β) —were measured at the end of the treatment. When rats were given different concentrations of residual aqueous fraction, there was a significant (*p* < 0.05) reduction in blood glucose, malondialdehyde (MDA), IL-1β, and TNF-α levels, as well as a significant (*p <* 0.05) increase in SOD (superoxide dismutase), catalase and insulin levels when compared to the diabetic control group. Furthermore, the 400 mg/kg body weight dosage concentration was found to be the most effective. This result suggests that the residual aqueous fraction of *Ethulia conyzoides* possesses significant antidiabetic, antioxidant and anti-inflammatory activities.

**Highlights**
- Sub-acute antidiabetic studies were done with varying doses (100, 200, and 400 mg/kg body weight). Treatment with the highest dose of residual aqueous fraction (RAF) of *Ethulia conyzoides* caused a 67.13% reduction in the blood glucose level of the diabetic rats.
- In-vivo antioxidant studies revealed that serum SOD and catalase levels in diabetes groups treated with the residual aqueous fraction of *Ethulia conyzoides* increased significantly (*p* < 0.05), while serum MDA levels decreased significantly (*p* < 0.05) when compared to the diabetic untreated group.
- The highest dose of 400 mg/kg b.w. was found to be the most effective, and treatment with 400 mg/kg b.w of *Ethulia conyzoides* residual aqueous fraction caused a 30.80% and 63% reduction in TNF-α and IL-1β, respectively.
- The RAF of *Ethulia conyzoides* has ameliorative effects for type 2 diabetes (T2D).

## INTRODUCTION

Diabetes, a metabolic disorder, is regarded as one of the most serious global health issues, afflicting both young and old people worldwide, regardless of gender. (Animaw & Seyoum 2017; Karuranga *et al*. 2019). According to the International Diabetes Federation, the number of people living with diabetes has increased from 151 million in 2000 to approximately 463 million in 2019, with a projected increase to 700 million by 2045 (Karuranga *et al*. 2019). In Africa, about 19 million adults aged 20 years to 79 years had diabetes in 2019, and this figure is likely to rise to about 47 million by 2045; while in Nigeria, about 2.7 million people (aged 20–79) were living with diabetes (Karuranga *et al*. 2019).

Chronic inflammation has been linked to insulin resistance and type 2 diabetes pathogenesis, according to research (Barzilay *et al*. 2001; Duncan *et al*. 2003). Badawi *et al*. (2010) and Wang *et al*. (2013) discovered that several inflammatory markers are involved in type 2 diabetes. Moreover, elevated plasma concentrations of pro-inflammatory cytokines such as interleukin-1β (IL-1β) and tumor necrosis factor-α (TNF-α) have been linked to insulin resistance and are found in obesity, metabolic syndrome and type 2 diabetes (Nilsson *et al*. 1998; Spranger *et al*. 2003).

The use of medicinal plants has been part of the history of mankind (Gidey *et al*. 2015; Odhiambo *et al*. 2011). There is high reliability among people in Africa on their continuous use because of the belief that they are the most effective ways of treating diverse diseases (Joshi & Joshi 2000). *Ethulia conyzoides Linn (Asteraceae)* is an herb that can grow up to 1.5 m tall in wet grassland or along a river. The leaves are used as therapy for cancer in Madagascar (Burkill 1985) and South Western Nigeria (Sowemimo *et al*. 2009). The plant is a source of natural antioxidants (Aliyu *et al*. 2012). It is an anti-helminthic for round worms and abdominal disorders; it is used to treat headaches and dysmenorrhea; and crude methanol extract of *E.conyzoides* aerial parts has antibacterial activity (El-Bassuony 2009; Noumi *et al*. 1999). It has been reported that extracts of *E. conyzoides* contains flavonoids, triterpenoids and sterols (Mahmoud *et al*. 1983) which have been said to be responsible for their anti-diabetic effect (Gaikwad *et al*. 2014). An interview with people living in Okpokwu Local Government Area of Benue State claimed that they have been using *E. conyzoides* to treat diabetic cases, hence the need to explore its potential. We investigated the acute effect of different doses of residual aqueous fraction on insulin, antioxidant, and cytokine levels in this study.

## MATERIALS AND METHOD

### Chemicals and Reagents

Streptozotocin (STZ) was procured from Sigma Aldrich (United State of America, USA); fructose (Kem Light Laboratories PVT Ltd, India); Simas margarine (PT Salim Ivomas Pratama Tbk, Indonesia); normal diet feed (Grand Cereals Limited, Jos, Nigeria); rat insulin ELISA kit (Fine Test Kit, Wuhan, China); and all other chemicals and reagents used were of analytical grade and procured from appropriate manufacturing companies.

### Plant Material

The whole plant of *E. conyzoides* Linn was harvested from its natural habitat at the end of the raining season at Okpokwu L.G.A. of Benue State. It was identified by Mr. Namadi Sanusi at the Herbarium Unit of the Department of Botany, Faculty of Life Sciences, Ahmadu Bello University (A.B.U), Zaria, Nigeria, with a specimen voucher number of 7098 previously deposited in the herbarium.

### Experimental Animals

The total of 42 apparently healthy male Wistar rats weighing 120 g–150 g, purchased from the Animal House of the Department of Pharmacology and Therapeutics, A.B.U Zaria, were kept in well-aerated cages, given access to animal feed and water *ad libitum*, allowed to acclimatize for 2 weeks, and then maintained under standardised environmental conditions (22°C–28°C, 60%–70% relative humidity, and a 12 h dark light cycle). Ethical clearance was obtained from the A.B.U Committee on Animal Use and Care (approval number: ABUCAUC/2019/007). All institutional guidelines for experimental protocol were adhered to, as was strict compliance with national and international laws and guidelines for care and use of laboratory animals in research.

### Methods

#### Preparation and extraction of plant material

The plant sample was rinsed in clean water to remove debris and dust particles, then air dried at room temperature. The dried whole plant sample was ground into powder using a mortar and pestle. About 1700 g of the grounded sample was suspended in 70% crude methanol (1:10 *w/v*) for 48 h at room temperature with frequent agitation (cold maceration). The mixture was filtered off using a Whatman filter paper number 1 (1 mm mesh sieve), and the methanol solvent in the filtrate was evaporated completely using a rotary evaporator at 40°C. The sample was then concentrated by drying in a water bath maintained at a temperature of 45°C to obtain dried extract. The solvent-free crude methanol extract was kept in a sealed sample bottle and refrigerated at 2°C–4°C until further use was required (Otsuka 2006).

#### Partitioning of the crude methanol extract of E. conyzoides

The solvent-free crude methanol extract (122 g) was suspended in 50 mL of distilled water and then partitioned with n-hexane and ethyl-acetate consecutively to obtain n-hexane fraction, the ethyl-acetate fraction and the residual aqueous fraction. n-Hexane was added to the crude methanol extract that was dissolved in distilled water. It was then turned into a separating funnel, shaken, and allowed to stand for phase separation into two fractions. The n-hexane fraction was carefully decanted after partitioning, then more of the n-hexane solvent was added, and the same process was repeated several times until it was completely partitioned to obtain the n-hexane fraction. The same process above was repeated using ethyl acetate as the solvent to obtain the ethyl acetate fraction. The resulting residue was dissolved in water and referred to as the residual aqueous fraction. Each fraction obtained was concentrated using a rotary evaporator, and the remaining solvent in the extract was allowed to evaporate at room temperature to a constant weight (Otsuka 2006). The process of fractionation (partitioning) is a purification step for the crude extract.

#### Induction of type 2 diabetes

The induction of type 2 diabetes was carried out as described by Okoduwa *et al*. (2017a) with modifications. Animal feed (pelleted broiler finisher; Vital feed brand) was fortified with margarine in a ratio of 10 g of animal feed to one gramme of margarine. This was administered, along with 20% fructose solution as drinking water, to the rats *ad libitum* for six weeks. They were then fasted overnight and injected intraperitoneally with dissolved streptozotocin (STZ) (in a citrate buffer pH 4.5) at a single low dose of 45 mg/kg body weight. The first 24 h after induction, the animals were given a 5% glucose solution as drinking water.

#### Confirmation of diabetes

This was done ten days after STZ induction using a glucose test strip and glucometer on blood samples obtained from rats via tail puncture. Following confirmation, diabetic animals with fasting blood glucose (FBG) ≥ 200 mg/dL, Homeostatic Model Assessment of Insulin Resistance (HOMA IR) > 5, and Homeostatic Model Assessment of β-cell (HOMA-β) < 200 were included in the study (Okoduwa *et al*. 2017b; Srinivasan *et al*. 2005).

#### Animal grouping

The rats were divided into seven groups of six rats each, and the treatment was administered for 21 days.

Normal Control: Normal rats without induction and treatment

Diabetic Control: Diabetic rats without treatment

Diabetic rats treated with 500 mg/kg b.w Metformin

Diabetic rats treated with 100 mg/kg b.w residual aqueous fraction of *E. conyzoides*

Diabetic rats treated with 200 mg/kg b.w residual aqueous fraction of *E. conyzoides*

Diabetic rats treated with 400 mg/kg b.w residual aqueous fraction of *E. conyzoides*

Normal rats treated with 400 mg/kg b.w residual aqueous fraction of *E. conyzoides*

The fraction was administered to the animals orally.

Weekly body weight change was measured during the entire experimental period. The percentage body weight (b.w) was calculated using this formula:


$$\%\;\text{change in b. w}=\frac{\text{final b.w}-\text{initial b.w}}{\text{initial b.w}}\times 100$$

Also, the percentage feed and fluid intake were extrapolated using this formula:


$$\text{Percentage change in feed}/\text{fluid intake}=\frac{\text{Initial weight}/\text{volume of feed}/\text{fluid}-\text{leftover weight}/\text{volume of feed}/\text{fluid}}{\text{Initial weight}/\text{volume of feed}/\text{fluid}}\times 100$$

### Sample Collection

After 21 days of treatment, the animals were fasted overnight, anaesthetised using chloroform and then sacrificed by decapitation. Blood was collected into plain bottles and was placed immediately on ice for 3 h, then centrifuged at 3000 rpm for 15 min to obtain the serum used for biochemical findings. The liver from the control and experimental groups of the rats were excised and rinsed with cold saline. The preparation of liver homogenate was done by homogenising 1 g of liver in 4 mL of 0.1M phosphate buffer saline at pH 7.4. The homogenates were centrifuged at 3,000 rpm for 15 min. The supernatant was collected as liver tissue homogenate, and was used for the *in-vivo* antioxidant activity.

#### Determination of in-vivo antioxidant, insulin and cytokines activity

Superoxide dismutase (SOD) was determined by a method described by Fridovich (1989). Catalase activity was determined as described by Sinha (1972). Lipid peroxidation was assessed by thiobarbituric acid reactive substances Determination (TBARS) formation (Ohkawa *et al*. 1979). Insulin, TNF-α and IL-β were measured using ELISA assay kits as directed by the manufacturer.

### Insulin Sensitivity, Resistance and **β**-cell Function Estimation


$$\begin{array}{l} \text{Insulin sensitivity}=\frac{1}{\text{Log }\left\{\text{Fasting serum insulin }\left(\frac{\text{U}}{\text{L}}\right)\right\}\times \text{Log }\left\{\text{Fasting blood glucos }\left(\frac{\text{mmol}}{\text{L}}\right)\right\}}\\ \text{HOMA-IR}=\;\left\{\text{Fasting serum insulin }\left(\frac{\text{U}}{\text{L}}\right)\times \text{Fasting blood glucose }\left(\frac{\text{mmol}}{\text{L}}\right)\right\}/22.5\\ \text{HOMA-}\beta =\;\frac{20\times \text{Fasting serum insulin }\left(\frac{\text{U}}{\text{L}}\right)}{\text{Fasting blood glucos }\left(\frac{\text{mmol}}{\text{L}}\right)}-3.5\\ \text{Conversion factor:}\text{ Insulin }(1\text{U}/\text{L}=7.174\;\text{pmol}/\text{L})\;\text{and blood glucose }(1\;\text{mmoL}/\text{L}=\;18\;\text{mg}/\text{dl}). \end{array}$$

### Statistical Analysis

All statistical analyses were conducted using the statistical package for the social sciences (SPSS program version 25.0). The outcomes are presented as mean ± standard deviation (SD). The data were analysed by the one-way analysis of variance (ANOVA) and repeated-measure ANOVA where necessary. The Duncan multiple-range test was used to determine the level of significance. *P* value less than 0.05 was considered as significant (*p* < 0.05).

## RESULTS

### Effect of Residual Aqueous Fraction of *E. conyzoides* on Mean Fluid (mL/rat/day) and **Feed intake (g/rat/day)** of Induced Type 2 Diabetic Rats for the 21 days of Treatment

**Figure 1:**
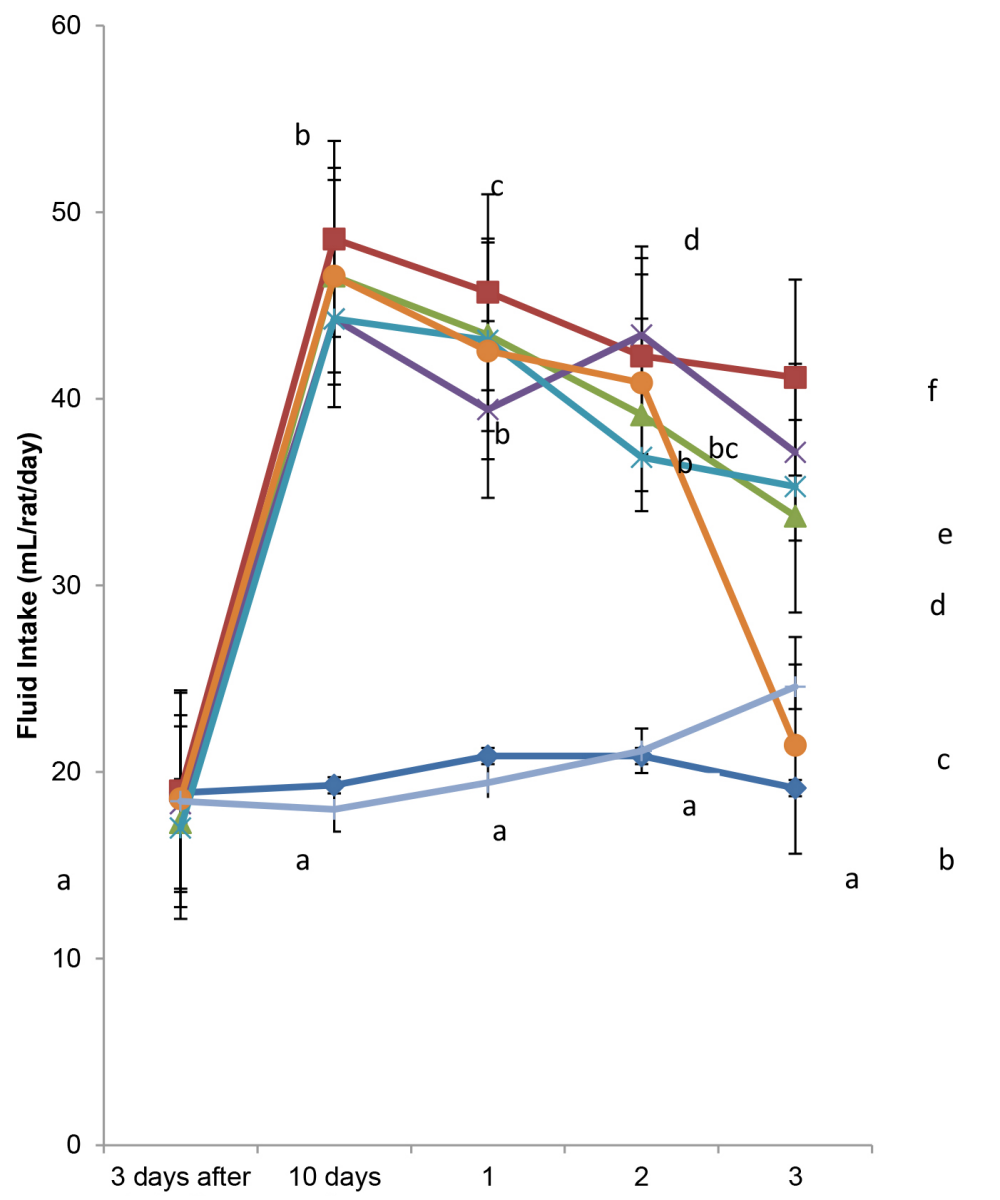
Effect of the residual aqueous fraction of *E. conyzoides* on the mean fluid intake (mL/rat/day) of induced type 2 diabetic rats for the 21 days of treatment.

**Figure 2:**
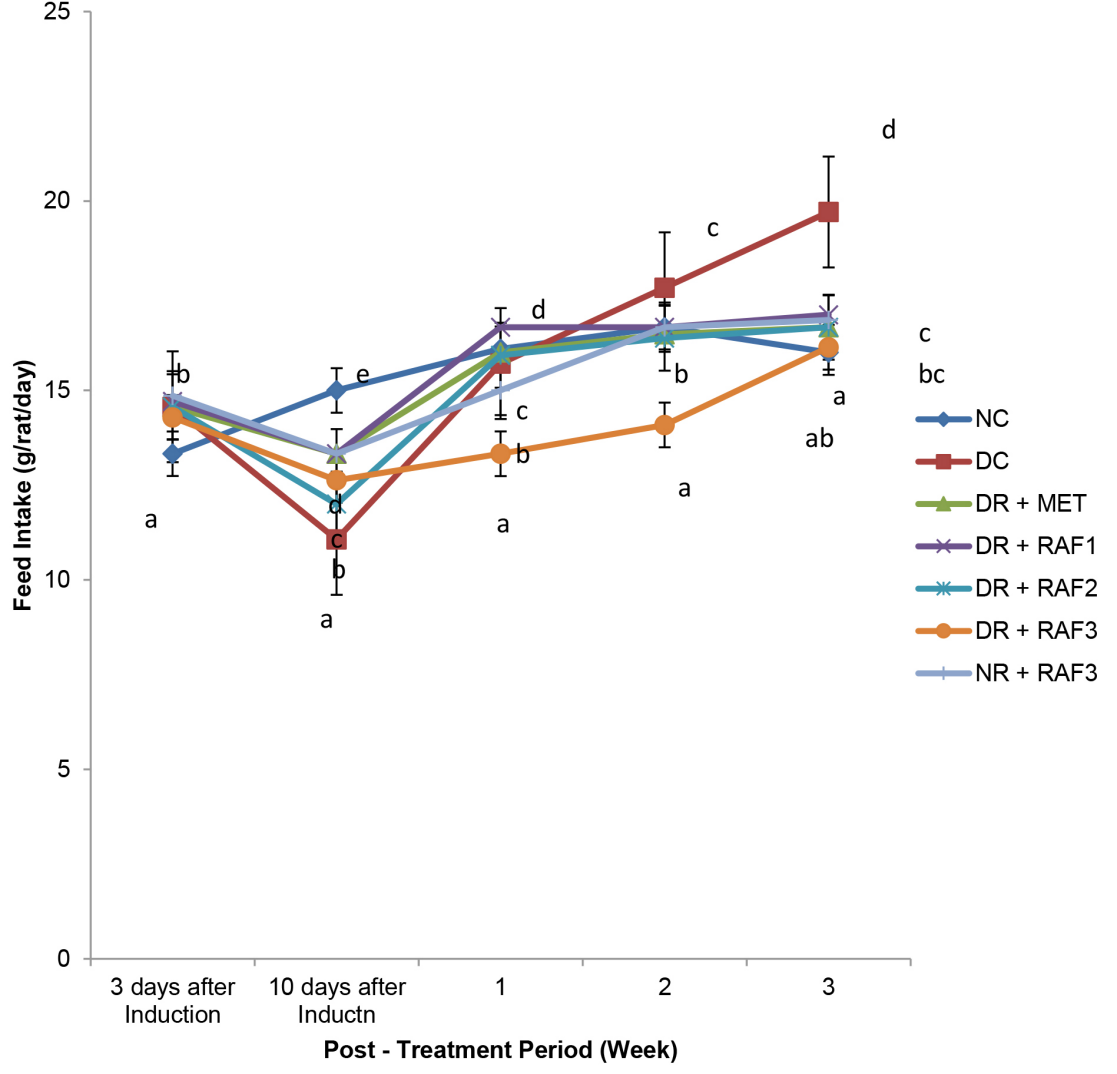
Effect of the residual aqueous fraction of *E. conyzoides* on the mean feed intake (g/rat/day) of induced type 2 diabetic rats for the 21 days of treatment.

The mean fluid and feed intake of each experimental animal on a daily basis throughout the experimental duration is depicted in Figs. 1 and 2, respectively.

At the induction, the diabetic groups had an increased fluid intake compared to the normal group, although it was not significant (*p* > 0.05). Fluid intake was significantly (*p* < 0.05) higher in the diabetic untreated group during the week of diabetes confirmation, as well as in weeks 2 and 3.

The diabetic groups at induction had a significant (*p* < 0.05) increase in feed intake compared to the normal group. Whereas the diabetic untreated group had a significant (*p* < 0.05) decrease in feed intake during the week that the diabetes was confirmed. Meanwhile, in the diabetic control group, feed intake increased significantly (*p* < 0.05) at weeks 2 and 3 compared to all other groups.

### Effect of Residual Aqueous Fraction of *E. conyzoides* on the Body Weight of Induced Type 2 Diabetic Rats for 21 Days of Treatment

**Figure 3:**
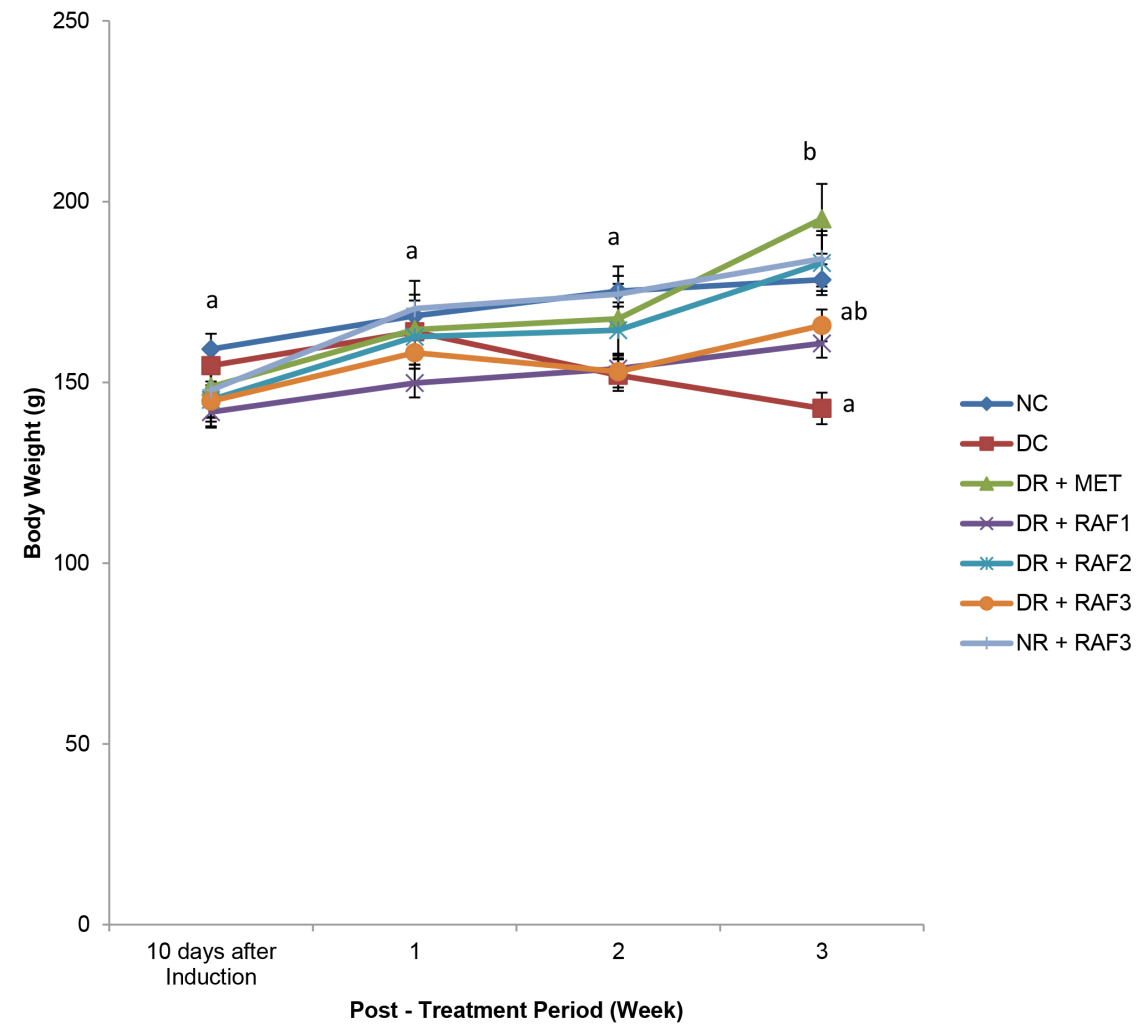
Effect of residual aqueous fraction of *E. conyzoides* on the body weight of induced Type 2 diabetic rats for 21 days of treatment.

The result (Fig. 3) shows that diabetic untreated rats had a significant (*p* < 0.05) reduction in body weight change compared with normal control.

Treatment with the standard drug (metformin) and different concentrations of the residual aqueous fraction of *E. conyzoides* increased body weight significantly (*p* < 0.05). The rat group treated with 500 mg/kg b.w of metformin, 200 mg/kg b.w residual aqueous fraction, and 400 mg/kg b.w residual aqueous fraction showed a significant (*p* < 0.05) increase in body weight.

### Effect of Residual Aqueous Fraction of *E. conyzoides* on the Weekly Percentage Change in Blood Glucose Level of Induced Type 2 Diabetic Rats for 21 Days of Treatment

The initial blood glucose of all the diabetic rats was higher than the normal control rats. Treatment with different doses (100 mg/kg b.w., 200 mg/kg b.w and 400 mg/kg b.w of residual aqueous fraction of *E. conyzoides* significantly (*p* < 0.05) lowered the blood glucose level of the diabetic rats (Table 1).

The rat group treated with 400 mg/kg b.w residual aqueous fraction showed the highest percentage (−73.70%) reduction in blood glucose level. The rat group treated with 100 mg/kg b.w had the least percentage (−28.42%) reduction. The result also revealed that the induced treated groups are dose dependent.

### Effect of Residual Aqueous Fraction of *E. conyzoides* on Weekly Blood Glucose Level of Induced Type 2 Diabetic Rats for 21 Days of Treatment

**Figure 4:**
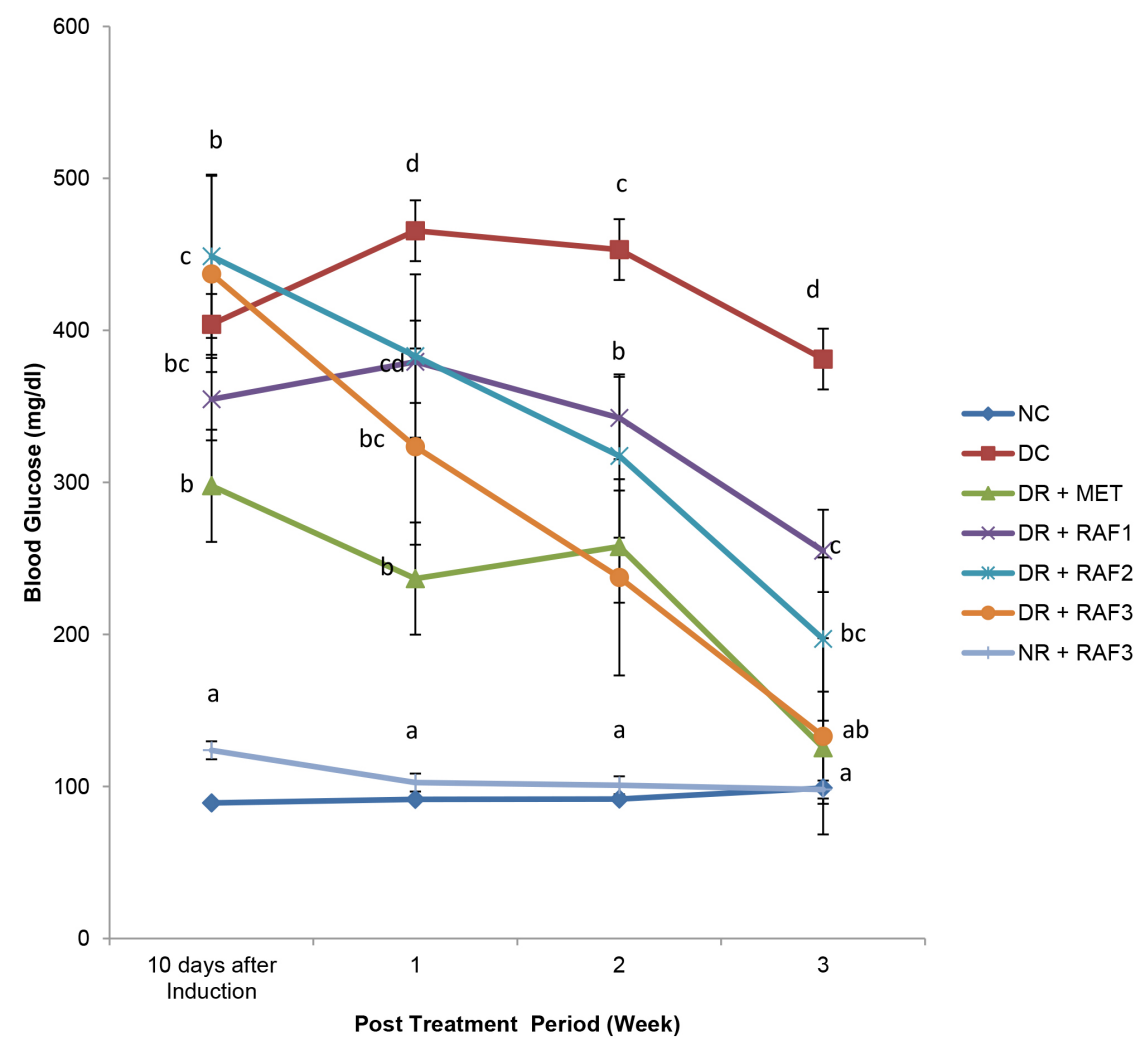
Effect of residual aqueous fraction of *E. conyzoides* on weekly blood glucose level of induced type 2 diabetic rats for 21 days of treatment.

There was a significant (*p* < 0.05) increase in the blood glucose level after the induction of diabetes (Fig. 4). Upon treatment, a gradual decrease in the blood glucose level was observed among the diabetic treated group when compared to the normal control and diabetic control group.

### Effect of Residual Aqueous Fraction of *E. conyzoides* on the Fasting Blood Glucose, Insulin Sensitivity, Resistance and **β**-cell Function of Induced Type 2 Diabetic Rats after 21 Days of Treatment

The calculated insulin sensitivity index, HOMA-IR and HOMA-β showed that HOMA-IR index was significantly (*p* < 0.05) higher in the diabetic control group when compared to the other groups while the insulin sensitivity and HOMA-β cell functioning index were significantly (*p* < 0.05) lower in the diabetic control group when compared to the other groups (Table 2).

### Effects of Residual Aqueous Fraction of *E. conyzoides* on Liver SOD, Catalase and MDA of Induced Type 2 Diabetic Rats for 21 Days of treatment

SOD, catalase and MDA levels show that the induction of diabetes significantly (*p* < 0.05) decreased SOD and catalase and significantly (*p* < 0.05) increased MDA levels (Table 3).

The different concentrations of *E. conyzoides* significantly (*p* < 0.05) increased SOD and catalase activities, while those of MDA were significantly (*p* < 0.05) reduced compared to diabetic control. Normal rats treated with 400 mg/kg b.w residual aqueous fraction of *E. conyzoides* had significantly (*p* < 0.05) higher SOD and catalase activities and significantly (*p* < 0.05) lower MDA activity compared with all treated groups. This result also revealed that the diabetic treatment groups are dose dependent.

### Effect of Residual Aqueous Fraction of *E. conyzoides* on the TNF-**α** and IL-1**β** Levels of Induced Type 2 Diabetic Rats after 21 Days of Treatment

The diabetic untreated group shows significant (*p* < 0.05) increase in the levels of TNF-α and IL-1β when compared to normal group (Table 4). Upon treatment, the diabetic rats treated with the different doses of residual aqueous fraction had significant (*p* < 0.05) decrease in the levels of TNF-α and IL-1β in the rats especially the group treated with the highest dose of residual aqueous fraction.

## DISCUSSION

The onset of type 2 diabetes (T2D) is strongly associated with insulin resistance and pancreatic β-cell dysfunction (WHO 2013). The significant increase (*p* < 0.05) in food intake (polyphagia), excessive fluid intake (polydipsia), and reduction in body weight observed in the diabetic control rats in this study are characteristics of T2D. These characteristics were improved by using residual aqueous fraction. From literature, Yerima and Samaila (2018) also found out that the residual aqueous fraction of their medicinal plant could be used in the treatment of diabetes, as the fraction significantly (*p* < 0.05) dropped the level of blood glucose in the diabetic rats.

High fluid intake was seen in T2D rats; the reduction in fluid intake seen in the T2D treated rats could be as a result of increased intracellular water, which triggers the osmoreceptor of the thirst centre of the brain, leading to less water intake (Okoduwa *et al*. 2017c).

Weight reduction is key to the prevention and management of type 2 diabetes in the obese (Inzucchi *et al*. 2015). There was a significant (*p* < 0.05) reduction in body weight observed in the diabetic control, and this could be due to a decrease in appetite, feed intake, or increase in the catabolic effect, which is evident in T2D (Russell *et al*. 2001). However, all the groups treated with residual aqueous fraction especially the group treated with the highest dose showed significant (*p* < 0.05) improvement in body weight and this indicates that residual aqueous fraction of *E. conyzoides* may be able to ameliorate hyperglycaemia-induced muscle wastage; this is in line with the work of Petchi *et al*. (2014) which found out that a combination of three different plant extracts significantly improved the body weight in diabetic group treated with the combination.

Insulin is a hormone needed by the cell for the uptake of glucose (Qaid & Abdelrahman 2016). The level of insulin in the plasma conveys a signal indicating the adiposity grade to any insulin-sensitive tissue. The residual aqueous fraction of *E. conyzoides* could be said to have exerted its anti-diabetic activity through its ability to decrease insulin resistance and improve the sensitivity of the cells and tissues to endogenous insulin, as seen by the decreased blood glucose level. This is in line with the work of Zhang *et al*. (2016), who reported that T2D rats administered polysaccharides from *Pleurotus ostreatus* for four weeks showed a significant (*p* < 0.05) decrease in insulin resistance.

Hyperglycemia results in free radical formation through various biochemical reactions. Bajaj and Khan (2012) discuss how this causes lipid peroxidation, which causes the tissue damage seen in diabetes (Raza *et al*. 2011). The significant (*p* < 0.05) decrease in MDA levels in the liver of diabetic-induced treated group compared to the diabetic control group in this study suggests that treatment with residual aqueous fractions of *E. conyzoides* may exert antioxidant activities, reduce hyperglycemia, and protect the tissue from lipid peroxidation. This is similar to the report of Kumawat *et al*. (2013), who found that MDA levels were significantly (*p* < 0.05) increased in T2D with or without nephropathy as compared to controls.

The therapeutic potentials of plants have been related with their antioxidant potentials (Eleazu *et al*. 2011). Endogenous antioxidant enzymes like catalase and superoxide dismutase (SOD) are body defence mechanisms to prevent and neutralise free radical-induced damage. The decreased activity of SOD and catalase in the liver tissues of T2D rats may be due to the free radicals generated by the Streptozotocin (Srinivasan & Pari 2012; Szkudelski 2001). When diabetic treated rats were compared to diabetic control rats, there was a significant (*p* < 0.05) increase in superoxide dismutase and catalase. This indicates that the residual aqueous fraction of *E. conyzoides* contains free radical scavenging activity, which could exert a beneficial action against pathophysiological alterations caused by the presence of superoxide and hydroxide radicals. Daryoush *et al*. (2011) reported that a reduction in SOD activity is a sensitive guide to hepatocellular damage and is the most sensitive enzymatic index in liver injury.

Oxidative stress mirrors the disparity between the generation of reactive oxygen species and the antioxidant defense system in the body (Sies 2015). It contributes to the progression of T2D by enhancing the secretion of pro-inflammatory cytokines like TNF-α and IL-1β. In this study, the decrease in the levels of TNF-α and IL-1β in diabetic treated group compared to the diabetic untreated group could be as a result of decrease in blood glucose level and this may have contributed to the observed decrease in the level of TNF-α and IL-1β. This is in agreement with the research done by Hämäläinen *et al*. (2007), which showed that antioxidant phytochemicals in plant extract inhibit inflammation by inhibiting nuclear factor kappa beta (NF-kB) activations. Down regulation of NF-κB, which acts as a potent transcription factor in initiating inflammation, may represent a possible mechanism to inhibit T2D.

It can be proposed as the putative mode of action that the residual aqueous fraction of *E. conyzoides* exerts anti-diabetic potential via its antioxidant and anti-inflammatory effects. The standard drug (metformin) used acts by lowering both basal and postprandial plasma glucose. It reduces hepatic glucose production, lowers intestinal glucose absorption, and improves insulin sensitivity by increasing peripheral glucose uptake and utilisation (Rena *et al*. 2017); so this extract too, may have worked through a similar mode of action as metformin.

## CONCLUSION

T2D is an important research topic for both clinicians and researchers. In this issue, we discussed how the residual aqueous fraction of *E. conyzoides* has modulatory potential against some of the metabolite derangements seen in diabetes, including elevated levels of oxidative stress marker (MDA), IL-1β and TNF-α. We hope this provides useful information to basic science researchers to catalyze novel therapeutic approaches and future research directions.

## Figures and Tables

**Table 1:** Effect of residual aqueous fraction of *E. conyzoides* on the weekly percentage change in the blood glucose level of induced type 2 diabetic rats for 21 days of treatment.

Groups	Weekly percentage change in the blood glucose level during treatment		

	Week 1	Week 2	Week 3
NC	2.67 ± 6.37^ab^	2.75 ± 13.56^ab^	11.01 ± 5.23^d^
DC	24.26 ± 27.85^b^	30.61 ± 62.73^b^	6.60 ± 38.42^cd^
DR + MET	−21.32 ± 37.29^a^	−14.41 ± 44.11^ab^	−66.52 ± 19.31^a^
DR + RAF1	6.66 ± 30.46^ab^	−3.95 ± 19.23^ab^	−28.42 ± 13.88^b^
DR + RAF2	−14.12 ± 8.10^a^	−29.20 ± 23.21^a^	−64.06 ± 34.94^a^
DR + RAF3	−23.81 ± 21.22^a^	−43.59 ± 14.38^a^	−73.70 ± 16.60^a^
NR + RAF3	−16.34 ± 8.60^a^	−17.82 ± 9.56^a^	−20.34 ± 10.83^bc^

*Notes*: Values are expressed as mean ± Standard Deviation n = 5 (reduction from 6 to 5 as a result of mortality); Values with different superscripts down the column are significantly different (p < 0.05). NC: Normal Control rats; DC: Diabetic control rats; DR: Diabetic rats; DR + MET: Diabetic rats + 500 mg/kg Metformin (standard drug); DR + RAF1: Diabetic rats + 100 mg/kg b.w residual aqueous fraction of E. conyzoides; DR + RAF2: Diabetic rats + 200 mg/kg b.w residual aqueous fraction of E. conyzoides; DR + RAF3: Diabetic rats + 400 mg/kg b.w residual aqueous fraction of E. conyzoides; NR + RAF3: Normal rats + 400mg/kg b.w residual aqueous fraction of E. conyzoides

**Table 2:** Effect of residual aqueous fraction of *E. conyzoides* on the fasting blood glucose, insulin sensitivity, resistance and β-cell function of induced type 2 diabetic rats after 21 days of treatment.

Groups	Fasting Blood Glucose (mg/dl)	HOMA-IR	HOMA-β	Insulin Sensitivity
NC	99.00 ± 4.58^a^	0.90 ± 0.07^a^	9.80 ± 0.74^bc^	2.39 ± 0.11^d^
DC	381.20 ± 81.34^c^	5.46 ± 1.67^c^	2.20 ± 1.37^a^	1.00 ± 0.09^a^
DR + MET	125.50 ± 26.35^a^	1.80 ± 0.37^a^	13.76 ± 3.28^c^	1.57 ± 0.15^b^
DR + RAF1	255.00 ± 56.92^b^	4.07 ± 0.80^bc^	6.17 ± 2.74^ab^	1.08 ± 0.07^a^
DR + RAF2	222.00 ± 91.98^b^	2.53 ± 1.43^ab^	6.36 ± 5.76^ab^	1.50 ± 0.38^b^
DR + RAF3	133.00 ± 9.13^a^	2.06 ± 0.17^a^	13.42 ± 1.88^c^	1.45 ± 0.05^b^
NR + RAF3	98.00 ± 12.57^a^	1.14 ± 0.06^a^	13.65 ± 0.25^c^	2.02 ± 0.06^c^

*Notes*: Values are expressed as mean ± Standard Deviation n = 5 (reduction from 6 to 5 as a result of mortality); Values with different superscripts down the column are significantly different (*p* < 0.05). NC: Normal Control rats; DC: Diabetic control rats; DR: Diabetic rats; DR + MET: Diabetic rats + 500 mg/kg Metformin (standard drug); DR + RAF1: Diabetic rats + 100 mg/kg b.w residual aqueous fraction of *E. conyzoides;* DR + RAF2: Diabetic rats + 200 mg/kg b.w residual aqueous fraction of *E. conyzoides;* DR + RAF3: Diabetic rats + 400 mg/kg b.w residual aqueous fraction of *E. conyzoides;* NR + RAF3: Normal rats + 400mg/kg b.w residual aqueous fraction of *E. conyzoides*.

**Table 3:** Effects of residual aqueous fraction of *E. conyzoides* on liver SOD, catalase and MDA of induced type 2 diabetic rats for 21 days of treatment.

Groups	SOD (mmol/min/g of tissue)	Catalase (moles of H_2_O_2_/min/g of tissue)	MDA (μmol/mg protein)
NC	20.66 ± 1.61^e^	16.18 ± 0.58^de^	109.64 ± 2.91^b^
DC	11.2 ± 0.51^a^	9.38 ± 0.91^a^	129.60 ± 2.06^e^
DR + MET	16.64 ± 1.12^d^	13.75 ± 0.79^c^	116.80 ± 2.37^c^
DR + RAF1	12.88 ± 0.72^ab^	10.88 ± 0.45^b^	123.70 ± 4.49^d^
DR + RAF2	14.50 ± 1.47^bc^	13.08 ± 0.45^c^	121.50 ± 3.03^d^
DR + RAF3	16.20 ± 0.84^cd^	15.50 ± 0.63^d^	116.03 ± 2.61^c^
NR + RAF3	19.38 ± 1.88^e^	17.05 ± 0.83^e^	104.90 ± 2.99^a^

*Notes*: Values are expressed as mean ± Standard Deviation n = 5 (reduction from 6 to 5 as a result of mortality); Values with different superscripts down the column are significantly different (p < 0.05); NC: Normal Control rats; DC: Diabetic control rats; DR: Diabetic rats; DR + MET: Diabetic rats + 500 mg/kg b.w Metformin (standard drug); DR + RAF1: Diabetic rats + 100 mg/kg b.w residual aqueous fraction of *E. conyzoides;* DR + RAF2: Diabetic rats + 200 mg/kg b.w residual aqueous fraction of *E. conyzoides;* DR + RAF3: Diabetic rats + 400 mg/kg b.w residual aqueous fraction of *E. conyzoides;* NR + RAF3: Normal rats + 400 mg/kg b.w residual aqueous fraction of *E. conyzoides*.

**Table 4:** Effect of residual aqueous fraction of *E. conyzoides* on the TNF-α and IL-1β levels of induced type 2 diabetic rats after 21 days of treatment.

GROUP	TNF-α (pg/mL)	IL-1β (pg/mL)
NC	6.30 ± 0.82^a^	40.00 ± 3.08^a^
DC	29.75 ± 4.40^e^	74.33 ± 8.14^d^
DR + MET	11.27 ± 0.81^c^	41.67 ± 2.89^a^
DR + RAF1	20.55 ± 2.01^d^	56.25 ± 2.52^c^
DR + RAF2	21.07 ± 3.00^d^	52.67 ± 5.13^bc^
DR + RAF3	7.30 ± 1.32^ab^	45.67 ± 5.00^ab^
NR + RAF3	10.38 ± 1.52^bc^	45.00 ± 11.73^ab^

*Notes*: Values are expressed as mean ± Standard Deviation *n* = 5 (reduction from 6 to 5 as a result of mortality); Values with different superscripts down the column are significantly different (*p* < 0.05). NC: Normal Control rats; DC: Diabetic control rats; DR: Diabetic rats; DR + MET: Diabetic rats + 500 mg/kg b.w Metformin (standard drug); DR + RAF1: Diabetic rats + 100 mg/kg b.w residual aqueous fraction of *E. conyzoides;* DR + RAF2: Diabetic rats + 200 mg/kg b.w residual aqueous fraction of *E. conyzoides;* DR + RAF3: Diabetic rats + 400 mg/kg b.w residual aqueous fraction of *E. conyzoides;* NR + RAF3: Normal rats + 400 mg/kg b.w residual aqueous fraction of *E. conyzoides*.
